# Prediction of Needle Physiological Traits Using UAV Imagery for Breeding Selection of Slash Pine

**DOI:** 10.34133/plantphenomics.0028

**Published:** 2023-03-15

**Authors:** Xiaoyun Niu, Zhaoying Song, Cong Xu, Haoran Wu, Qifu Luan, Jingmin Jiang, Yanjie Li

**Affiliations:** ^1^ College of Landscape Architecture and Tourism, Hebei Agriculture University, Baoding 071000, China.; ^2^ Research Institute of Subtropical Forestry, Chinese Academy of Forestry, No. 73, Daqiao Road, Fuyang, Hangzhou 311400, Zhejiang Province, China.; ^3^ New Zealand School of Forestry, University of Canterbury, Private Bag 4800, 8041 Christchurch, New Zealand.

## Abstract

Leaf nitrogen (N) content and nonstructural carbohydrate (NSC) content are 2 important physiological indicators that reflect the growth state of trees. Rapid and accurate measurement of these 2 traits multitemporally enables dynamic monitoring of tree growth and efficient tree breeding selection. Traditional methods to monitor N and NSC are time-consuming, are mostly used on a small scale, and are nonrepeatable. In this paper, the performance of unmanned aerial vehicle multispectral imaging was evaluated over 11 months of 2021 on the estimation of canopy N and NSC contents from 383 slash pine trees. Four machine learning methods were compared to generate the optimal model for N and NSC prediction. In addition, the temporal scale of heritable variation for N and NSC was evaluated. The results show that the gradient boosting machine model yields the best prediction results on N and NSC, with *R*^2^ values of 0.60 and 0.65 on the validation set (20%), respectively. The heritability (*h*^2^) of all traits in 11 months ranged from 0 to 0.49, with the highest *h*^2^ for N and NSC found in July and March (0.26 and 0.49, respectively). Finally, 5 families with high N and NSC breeding values were selected. To the best of our knowledge, this is the first study to predict N and NSC contents in trees using time-series unmanned aerial vehicle multispectral imaging and estimating the genetic variation of N and NSC along a temporal scale, which provides more reliable information about the overall performance of families in a breeding program.

## Introduction

Carbohydrates, which are the primary products of photosynthesis in plants, provide a important energy source for plant growth, reproduction, and survival. In general, carbohydrates have been classified as structural carbohydrate or nonstructural carbohydrate (NSC). NSCs are a temporary storage of carbohydrates accumulated in trees when they are overproduced. Therefore, the variation in NSC concentration usually reflects the plant carbon availability and the overall carbon supply of plants [1]. Among them, the soluble sugar and starch contents in the form of sucrose account for more than 90% of the total NSC [2]. It has been reported that when the concentration of soluble sugar reaches a critical level, it will be converted to starch and stored; if the concentration drops below a critical level, then the starch can be converted back to soluble sugar for plant growth [3]. Nitrogen (N) is a component of amino acids and proteins in plants, as well as the primary component of chlorophyll, nucleic acids, various coenzymes, vitamins, and plant hormones. Insufficient N content levels could inhibit carbohydrate storage and hamper the transport of photosynthetic products [4]. For instance, N deficiency could reduce foliar photosynthesis [5] and cause more starch to accumulate in plant tissues, and too much starch stored in needles might also affect photosynthetic efficiency [6]. Therefore, the NSC and N contents in plant canopy leaves are important indicators that could reflect metabolic health, which is highly related to plant growth, development, and reproduction.

Traditional phenotypic analysis methodologies are time- and cost-consuming, which makes it challenging to meet the high-throughput and large sample size requirements of forest genetic breeding [7]. Unmanned aerial vehicle (UAV)-based remote sensing, with the advantages of being nondestructive, high throughput, and fast to collect, has become an efficient technique for measuring plant structure and monitoring canopy physiology content in agriculture and forestry [8].

Multitemporal UAV-based imagery with red, green, and blue (RGB) or multispectral sensors has been largely used to evaluate various plant traits in agriculture and forestry [9–11]. For example, multitemporal and multispectral UAV imagery can be used for biomass and N content prediction in legumes [12] and maize [13], sugar content prediction in sugarcane [14,15], and soluble sugar and starch content estimation in citrus leaves [16].

Partial least squares regression (PLSR) [17] and other machine learning algorithms, such as support vector machines (SVMs) [18], gradient boosting machines (GBMs) [19] and random forests (RFs) [20], have been widely used in a variety of remote sensing applications [21–23]. All of these methods generally require accurate chemical analysis of the target traits as a reference [24].

PLSR is frequently used to examine quantitative relationships between multiple predictors and response variables. It is used when there are few observations, a high proportion of missing data, and a high degree of correlation between predictors [25]. It has been reported that PLSR can be used to estimate the N content of field crops in real time [26–29]. SVM has been extensively used to solve regression problems using linear, polynomial, and spline networks in remote sensing [30,31]. GBM is a technique for combining a large number of decision trees to optimize predictive performance and minimize overfitting risks. It is a nonparametric method dealing with qualitative and quantitative variables, which account for nonlinear interactions between dependent and independent variables [32]. Belgiu et al. [20] proposed RF as an extension of the bagging classification tree. To improve the accuracy of the regression, RF quantified the importance of each variable and selected a candidate predictor. The model can also be used to deal with a large amount of high-dimensional data in the domains of remote sensing and artificial intelligence. It is a predictive ensemble model with different regression trees formed by random variables and selection of samples [33]. It is evaluated for accuracy by an error estimation technique (Out-Of-Bag error) [34,35]. It runs efficiently on large datasets with less impact by noise or overfitting [36], which can handle thousands of input variables without variable deletion and has fewer parameters than other machine learning algorithms, so it is commonly employed in remote sensing.

These regression models perform at various accuracies on different biochemical parameter inversions. For instance, the RF model performs better than the SVM model on the estimation of canopy leaf area index of apple trees [37], and PLSR is more stable and accurate than RF and SVM in terms of limited samples on the prediction of aboveground biomass using near-surface spectroscopy [38]. However, the SVM model yields better results than RF on the identification of leaf-scale wheat powdery mildew based on hyperspectral imaging [39].

Slash pine (*Pinus elliottii*) is a native American tree species mainly found in the southeastern United States. It has been widely planted in southern China since the 20th century [40]. It grows quickly, is drought- and flood-resistant, and is infertile. It is a good landscape tree and an economic tree due to its high yield of turpentine and wood [41]. However, it is also a typical coniferous tree that has large complex genomes, which makes the reference genomes of slash pine unavailable. Because of the lack of multiple time-series monitoring of slash pine and genetic research on canopy physiology, previous studies only focused on conventional genetic analysis using phenotypic data, such as tree height, diameter at breast height, and canopy area, to estimate biomass. As a result, slash pine remains in phenotypic genetic studies and analysis [42].

Our previous study showed a successful methodology for the detection of tree growth parameters and vegetation indices (VIs) using UAV multispectral imagery and used for genetic variation estimation in slash pine [43]. Although many achievements have been made in plant growth and leaf physiological prediction using UAV images for plant breeding, little is known about the use of multitemporal UAV imaging to predict canopy physiological features for breeding purposes in slash pine.

Hereafter, we combined multitemporal and multispectral data from 11 months of slash pine growth in 2021 with the aforementioned approach to achieving 3 main objectives: (a) to evaluate the prediction ability of the PLSR, SVR, GBM, and RF models on the canopy NSC and N contents; (b) to estimate the phenomic and genetic variation in canopy NSC and N in the time series; and (c) to enable genetic selection in the slash pine plantation based on various breeding purposes in the time series.

## Materials and Methods

### Study area and tree materials

Experiments were conducted in a slash pine breeding plantation site at Matou State Forest farm, Xuancheng, Anhui, China (30°45′N, 118°29′E). This site is a relatively flat land with an altitude difference of no more than 10 m. Seeds were collected from 20 open-pollinated families and planted in nurseries in 2011. Two years later, the seedlings were planted as single-tree plot designs using alpha lattice incomplete blocks. The plantation covers a total of 19 ha and is divided into 2 sites, each site containing 20 blocks and each block including 20 trees spaced 2 × 3 m apart. Each tree represents an individual family, and each block contains no duplicate family.

### UAV sensors and image acquisition

This study used a commercial Da-Jiang Innovations (DJI) Phantom 4 Multispectral UAV (DJI Company, Shenzhen, China) with 6 imaging sensors, including an RGB sensor and 5 multispectral sensors, each with 2.08 million effective pixels, to capture plantation images. The UAV multispectral camera weighs 1,487 g and has a resolution of 1,600 × 1,300 pixels and a sensor size of 4.87 × 3.96 mm (Table 1). The UAV is equipped with a real-time kinematic positioning system based on satellite navigation to improve position data precision. The positioning accuracies at vertical and horizontal are 1.5 cm + 1 ppm (1 ppm means that the error increases by 1 mm for every 1 km the aircraft moves) and 1 cm + 1 ppm, respectively. The multispectral images were acquired on the middle or late part of the day of each month in 2021 except February, with clear and windless or breezy weather being selected, including January 15, March 28, April 21, May 26, June 23, July 18, August 20, September 17, October 24, November 24, and December 28. The data acquisition time for each day was less than 2 hours.

**Table 1.** Spectral information and flight conditions of the DJI multispectral drone. FOV, field of view, in this study, it refers to the angle of the UAV lens to the ground.

**Band**	**Wavelength (nm)**	**FOV**	**Flight time**	10:00–12:00 AM
Blue	450	90°	**Date**	In every mid- month
Green	560			
Red	650	**Flight height**	**Weather**	Sunny Partly cloudy
Red-edge	730	30 m	**Wind**	At 1–3 m/s
Near-infrared	840			

### UAV image processing

The multitemporal images are preprocessed using the DJI terra software (version 3.4.4, DJI Company, Shenzhen, China) to create a 3-dimensional (3D) point cloud, a digital surface model (DSM; model depicting elevations of the tops of reflective surfaces), a digital terrain model (DTM; model depicting elevations of the terrain), and an orthomosaic image. The structure-from-motion and multiview-stereo photogrammetry generates 3D topographic models from a collection of ordinary digital images. It provides very high-resolution orthophoto mosaics in addition to topographic data, and its algorithm works by automatically recognizing and connecting point clouds on a set of overlapping photos (median of 70,000 key points per image). A self-calibrating bundle adjustment was used to create a sparse set of 3D point clouds after calibrating the camera settings for each image. MVS image-matching algorithms were then implemented to create a dense 3D point cloud [44–46]. Individual tree crown area was estimated from a 3D cloud model, and high-precision geo-position information was generated. Individual tree positions were detected and delineated using the variable-window-filter algorithm. The dense 3D point cloud was filtered, and DSM and DTM were produced, which were then used to orthorectify individual images and create an orthomosaic [47,48]. The canopy height model (CHM), which represents the potential height of each tree, is created in R software using the DSM and DTM raster GeoTIFF images, as shown in Eq. 1.

CHM=DSM−DTM (1)

CHMs were subsequently applied to detect all individual trees using the dalponte2016 function with a minimum height of 2.6 m and a maximum crown diameter of 2.5 m. The tree height and crown area of each detected individual tree were labeled manually with family, site, and block information, and tree crown polygons were generated from the crown area with the raster package [49]. Finally, the spectra of each individual tree were extracted using tree crown polygons.

### Vegetation indices

VIs are frequently used as a critical indicator for representing and interpreting vegetation growth. In general, VI values are used to monitor the growth and forecast the yields of crops [50]. We calculated 16 VIs as secondary traits to predict the NSC and N contents in the canopy of slash pine in this study (Table 2).

**Table 2.** Multispectral VI introduction: NIR, RE, R, G, and B denote the near-infrared, red-edge, red, green, and blue bands, respectively.

**Vegetation indices**	**Full name**	**Equation**	**Reference**
NDVI	Normalized difference vegetation index	(NIR − R) / (NIR + R)	[83]
GNDVI	Green normalized difference vegetation index	(NIR − G) / (NIR + G)	[84]
NDRE	Normalized difference red-edge	(NIR − RE) / (NIR + RE)	[85]
SAVI	Soil-adjusted vegetation index	1.5 * (NIR − R) / (NIR + R + 0.5)	[86]
OSAVI	Optimized soil-adjusted vegetation index	(NIR − R) / (NIR + R + 0.16)	[86]
RERVI	Red-edge ratio vegetation index	NIR / RE	[87]
LCI	Leaf chlorophyll index	(NIR − RE) / (NIR + R)	[88]
SCCCI	Simplified canopy chlorophyll content index	NDRE / NDVI	[89]
NRI	Nitrogen reflectance index	(G − R) / (G + R)	[90]
GRVI	Green–red vegetation index	(G − R) / (G + R)	[91]
MNLI	Modified nonlinear index	(NIR^2^ * 1.5 – G * 1.5) / (NIR^2^ + R + 0.5)	[92]
DVI	Difference vegetation index	NIR − R	[93]
EVI	Enhanced vegetation index	(NIR − R) / (1 + NIR + 6 * R − 7.5 * B)	[94]
RVI	Ratio vegetation index	NIR / R	[95]
NG	Norm green	G / (R + G + NIR)	[96]
NR	Norm red	R / (R + G + NIR)	[96]

### Ground data collection

In each month (11 months from 2021), during the flight task, 20 to 50 tree samples were randomly selected for needle collection. For each tree, pine needles were harvested from the north, south, west, and east sides of the top canopy with branch shears. They were mixed in plastic bags and labeled as a single tree canopy. Finally, the samples were collected and placed in a foam box with an ice pack. A total of 383 samples were collected and brought to the laboratory for the measurement of N and NSC. The location, block number, and family name of the slash pine samples were recorded to match with aerial imagery when the samples were taken. The collected needles were then sent to the Key Laboratory of Subtropical Forest Cultivation for the content detection of N and NSC.

### N measurement

The N content was measured using the Kjeldahl method [51,52]. After the slash pine needles were collected, they were placed in a dry bag for 30 min at 105° and were baked at 65° for 48 hours until a constant weight was reached. Each sample was mixed, ground to a powder and sieved. Using Na_2_SO_4_ and Se as catalysts, a 0.25-g sample was digested with 5 ml of concentrated H_2_SO_4_ at 380 °C until the sample solution was clear. After adjusting the volume of each digested sample to 50 ml with distilled water, 10 ml of the sample was distilled using a standard Kjeldahl process after adding 1 ml of a 40% NaOH solution. A distilled sample was titrated with H_2_SO_4_ standard solution until the end point was reached, and the N content was determined using Eq. 2:

$$N\;\text{content}\;\left(\%\right)=\frac{\left(\mathrm{Vs}-\mathrm{Vb}\right)\times N\times 0.014\times \mathrm{Vd}\times 100}{W\times \mathrm{Va}}$$ (2)

where *Vs* is the amount of a standard H_2_SO_4_ (ml) used for titration to reach the end point, *Vb* is the amount of a standard H_2_SO_4_ (ml) used for titration of the blank, *N* is the H_2_SO_4_ concentration, *Vd* is the amount of digested sample solution (ml), *W* is the weight of slashed pine needle samples (g), and *Va* is the amount of a sample solution for the analysis (10 ml).

### NSC measurement

NSCs were determined by the Anthrone method [53,54]. The 0.5-g slash pine needle samples were placed in a 25-ml cuvette supplemented with 10 ml of distilled water, and the mixture was allowed to stand at 100 °C for 1 hour before being filtered into 25-ml volumetric flasks. In a 7.5-ml reaction mixture, there were 0.5 ml of extracts, 0.5 ml of mixed reagent (1 g of anthrone + 50 ml of ethyl acetate), 5 ml of H_2_SO_4_ (98%), and 1.5 ml of distilled water. The mixture was heated at 100 °C for 1 min, and the absorbance read at 630 nm was the NSC content.

### Model application

The 16 VIs calculated 11 months of multispectral data and the N and NSC contents within the canopy measured in the laboratory were aggregated together and used to generate PLSR, RF, SVM, and GBM prediction models. The pls package [55] in R software was used to fit the PLSR model, and the number of components and validation for the PLSR model were set as 10 with one left out, respectively. The e1071 package [56] was used to fit the SVM and RF models, and there are 2 parameters that are important in the SVM calculation, including the penalty factor c and kernel parameter g. We used the grid search method for optimization and train function with svmRadial method. The RF model was established and validated using the randomForest function in the “randomForest” package [57] within the statistical software package R 4.2.0. The number of trees was set as 300 for the RF model, the most important parameter of RF [58]. The caret package [59] was used to fit the GBM model, and tuneGrid was used to train the model to obtain optimal value. In the current research, a GBM with a grid search method was used for the optimization of the learning rate and number of tree parameters. In addition, we used the variable importance function [varImp()] from the caret package after generating the GBM model to enable us to perform importance ranking of variables.

Among them, the data were split randomly into an 80% calibration set and a 20% validation set. The calibration set was used to build the calibration model, while the validation set was used to evaluate the predictive performance of the calibration model. The coefficient of determination (*R*^2^) and the root mean square error (RMSE) were used to quantify the model accuracy. The performance of the model was estimated by comparing the differences in *R*^2^ and RMSE between the estimated versus measured value plots.

### Estimation of genetic parameters

To estimate the genetic parameters underlying slash pine growth traits and spectral indices, a bivariate linear mixed model was fitted using restricted maximum likelihood. Details can be found in [60]. The model is represented by Eq. 3:

$$y=\mathrm{Xm}+Z_{1}b+Z_{2}f+e$$ (3)

where *y* denotes the response vector for tree traits and *X*, *Z*_1_, and *Z*_2_ denote the incidence matrices that connect observations to their corresponding effects. As a result, *m*, *b*, *f*, and *e* are the intercept, site, and random additive effect vectors for block, family, and residual effects, respectively. The narrow sense of *h*^2^ was estimated using variance components from the model as Eq. 4:

$$h_{i}^{2}=\frac{2.5\sigma_{\mathrm{fi}}^{2}}{\sigma_{\mathrm{fi}}^{2}+\sigma_{\mathrm{bi}}^{2}+\sigma_{\mathrm{ei}}^{2}}$$ (4)

where $\sigma_{\mathrm{fi}}^{2}$, $\;\sigma_{b_{i}}^{2}$ and $\;\sigma_{e_{i}}^{2}$ correspond to the family, block, and residual variances for the trait, respectively. Finally, the breeding values of canopy N and NSC content among different families of slash pine based on *h*^2^ were calculated, and the calculation formula is represented by Eq. 5:

$$\hat{a_{i}}=h_{i}^{2}\left(y_{i}-\hat{\mu }\right)$$ (5)

where $\hat{a_{i}}$ is the predicted breeding value of the slash pine families, $h_{i}^{2}$ is the heritability of the *i*th family, *y_i_* is the average observed value of the *i*th family, and $\hat{\mu }$ is the estimated value of the population mean.

All of the analyses were carried out with R software and the RStudio platform, which is available for free. The ggplot2 package [61] was used for visualization, and the raster package [49] was used for GeoTIFF image processing.

## Results

### Estimation and analysis of trees from UAV imaging

The calculation of tree height, the detection of individual trees, and the extraction of multispectral data for this research site have been described in detail by Song et al. [42] and Tao et al. [43] and will not be repeated here.

### Model selection

Table 3 shows the *R*^2^ and RMSE of the calibration and validation data for different prediction models of NSC and N. For the prediction accuracy of NSC, the GBM model yielded the highest *R*^2^ and lowest RMSE on the validation set, with values of 0.65 and 1.06%, respectively, followed by the SVM and RF models. The PLSR model yielded the lowest accuracy, with *R*^2^ and RMSE values of 0.55 and 1.13%, respectively. RF obtained the highest *R*^2^ and lowest RMSE on the calibration set, with values of 0.93 and 0.54%, respectively, but the accuracy was reduced to 0.59 for *R*^2^ and 1.17% for RMSE on the validation set.

**Table 3.** Comparison of the calibration set (Cal) and validation set (Val) *R*^2^ and RMSE between different prediction models of NSC and N.

	Model	Cal		Val	
		*R* ^2^	RMSE (%)	*R* ^2^	RMSE (%)
NSC	PLSR	0.56	1.16	0.55	1.13
	SVM	0.75	0.86	0.63	1.15
	GBM	0.72	0.97	0.65	1.06
	RF	0.93	0.54	0.59	1.17
N	PLSR	0.51	0.70	0.37	0.75
	SVM	0.51	0.68	0.48	0.83
	GBM	0.79	0.48	0.60	0.52
	RF	0.91	0.34	0.51	0.67

The prediction accuracies of all models on N were lower than the prediction on NSC.

The GBM model also yielded the highest *R*^2^ and lowest RMSE on the validation set for predicting N, with values of 0.60 and 0.52%, respectively, followed by RF and SVM. The PLSR model yielded the lowest accuracy, similar to the NSC prediction model, with *R*^2^ and RMSE values of 0.37 and 0.75%, respectively. Similar to the prediction of NSC, the RF still yielded the highest *R*^2^ and lowest RMSE on the calibration set, with values of 0.91 and 0.34%, respectively, but the accuracy was reduced to 0.51 for *R*^2^ and 0.67% for RMSE on the validation set. This suggests that overfitting was observed in RF for the prediction of both NSC and N. Overall, GBM showed the best accuracy for the prediction of N and NSC.

**Fig. 1.**
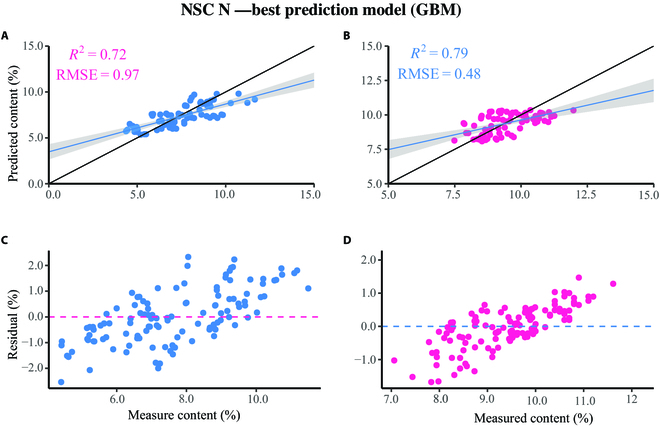
The connection between field-measured NSC and N contents and anticipated UAV data, where (A) and (C) are regression and residual graphs of NSC content, and (B) and (D) are regression and residual diagrams of N content.

Figure 1 shows the regression and residual plots for the GBM model for predicting NSC and N contents. Both predictive models tend to overestimate when the measured value is low. However, at higher measured values, predictive models tend to underestimate.

### Relationship between needle NSC and N contents and VIs

**Fig. 2.**
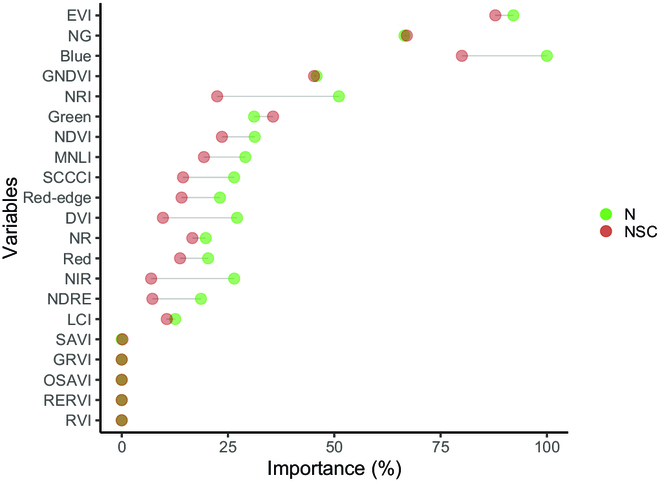
Ranking of 21 important variables used for modeling.

Figure 2 shows the ranking of all important variables used for GBM modeling. The importance of variable selection determines useful VIs in the modeling. Among them, enhanced vegetation index showed a high importance score in predicting both NSC and N contents, followed by norm green and green normalized difference vegetation index (NDVI). The blue band showed upward of 99% importance in estimating the N content while also reaching more than 75% importance in estimating the NSC content. The nitrogen reflectance index showed more than 50% importance in estimating the N content but only 20% importance in estimating the NSC content. The green band showed higher importance in NSC content than in N. NDVI, modified nonlinear index, simplified canopy chlorophyll content index, red-edge, DVI, norm red, Red, near infrared (NIR), normalized difference red-edge (NDRE), and leaf chlorophyll index showed significantly higher importance in predicting N content than NSC. However, the importance score of soil-adjusted vegetation index, green–red vegetation index, optimized soil-adjusted vegetation index, red-edge ratio vegetation index, and ratio vegetation index in estimating N content was 0.

### The variation in canopy C and N contents in multiple months

**Fig. 3.**
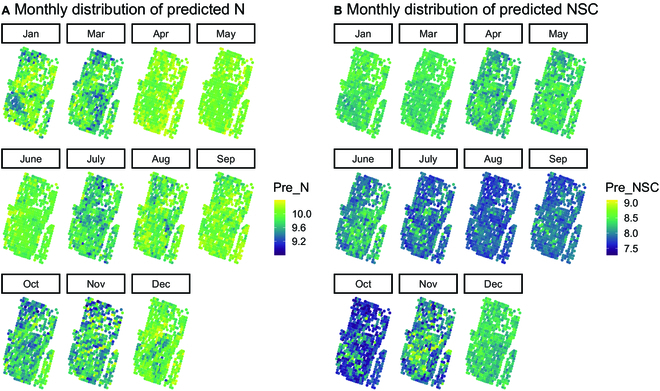
Monthly distribution of predicted N (A) and NSC (B) contents in each tree in the breeding plantation of slash pine in 2021. One point represents one tree.

**Fig. 4.**
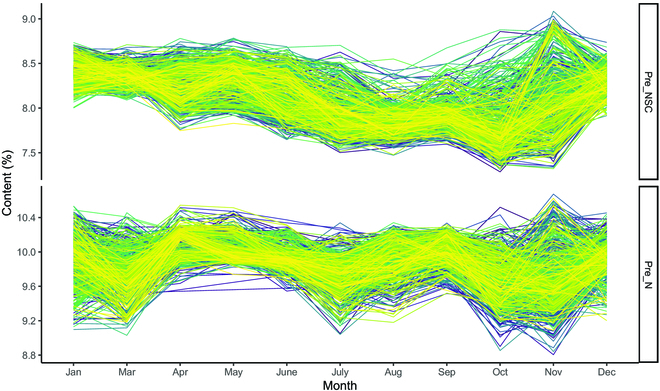
The monthly variation trend of NSC and N contents among different families in the slash pine breeding plantation. One line represents one family.

**Fig. 5.**
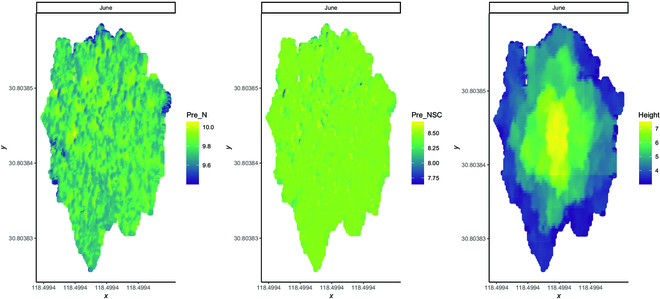
The distribution of predicted N and NSC contents within a tree canopy as an example tree.

Figure 3 depicts the monthly distribution of predicted NSC and N contents at the study site. The predicted N content (Fig. 3A) was higher in April, May, June, and September than in other months, while the predicted NSC content was the highest in January, March, and December (Fig. 3B). The monthly variation in predicted NSC and N contents among different families of slash pine in the study area is shown in Fig. 4. The predicted NSC content generally indicated a gradually decreasing trend from January to September and then an increasing trend from October to December. The lowest values were found in October for NSC at approximately 7.3%. The N content decreased in March, July, October, and November and increased in April, August, and September. The distribution of predicted N and NSC contents within the tree canopy in one example tree is plotted in Fig. 5. The predicted N content is primarily distributed on the new shoots from the canopy, and the predicted NSC content is evenly distributed throughout the canopy and is relatively lower than the predicted N content.

### Genetic variation and family selection

**Fig. 6.**
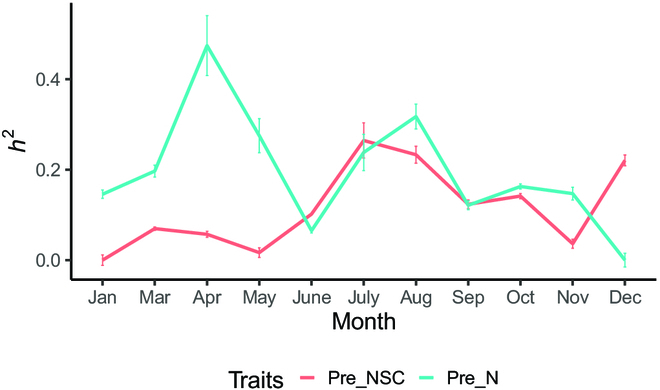
The variation in estimated *h*^2^ in predicted N and NSC from different months in 2021.

The variation in estimated *h*^2^ in predicted N and NSC from different months is displayed in Fig. 6. The *h*^2^ variation in the NSC content of the species from January to December ranged from 0 to 0.26. Among them are the overall trend of increase from January to July, a downward trend from July to November, and an upward trend after November. The highest value of *h*^2^ was 0.26 in July, and the lowest was 0 in January. The overall *h*^2^ of the N content was slightly higher than that of NSC, ranging from 0.01 to 0.49. The *h*^2^ with the highest N content appeared in April and August with 0.49 and 0.33, respectively, while the lowest *h*^2^ occurred in December with 0.01.

**Fig. 7.**
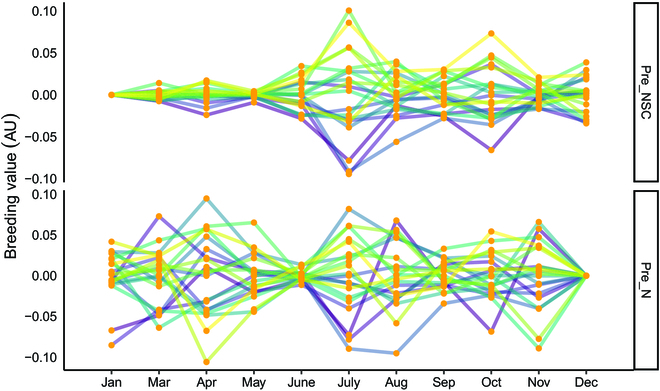
The variation in breeding values in predicted N and NSC contents from different months in 2021. Each line represents one individual family, and the total number of families is 20. AU, arbitrary units.

**Fig. 8.**
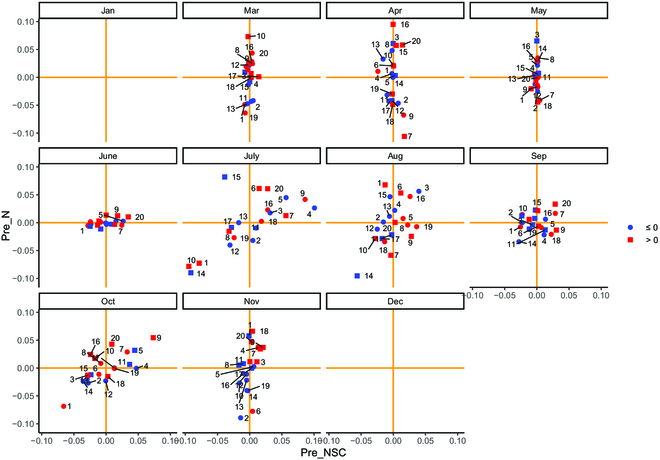
The relationship between the 2 traits (predicted NSC and N) breeding values of slash pine families in different months. The orange solid line is the average of the predicted NSC and N contents. The blue solid dots and the red solid square represent the breeding value of trees height (H) and crown area (CA) lower than the mean value and higher than the mean value, respectively. Pre_NSC: predicted NSC content; pre_N: predicted N content. The number of each point represents the family number.

The monthly variation in NSC and N content breeding value in different slash pine families is shown in Fig. 7. This result is roughly consistent with the *h*^2^ of each trait. The breeding values of NSC content in January, March, and May were low. The highest variant breeding values were found in July, ranging from 0.10% to −0.10%. The lowest breeding values occurred in December and June in predicting N content, with the lowest breeding value in December being 0. However, the breeding values showed substantial changes in April, July, and October, with the highest value reaching 0.10%. Different months showed varying breeding value distributions of slash pine based on the 2 traits (Fig. 8).

## Discussion

### Accuracy of prediction models

One of the most basic and widely used methods in remote sensing for assessing leaf physiological content is VIs. The selected VIs vary depending on the field of study and study material. For instance, Wang et al. [62] concluded that an exponential model with DVI as an independent variable could estimate the N content in maize leaves more accurately. Hassan et al. [63] utilized UAV multispectral technology to monitor VIs (NDVI and NDRE) throughout the growth cycle of wheat, accurately estimating the biomass and yield, as well as identifying the ideal period for high-yielding wheat genotypes. Similarly, using hyperspectral canopy reflectance data, a PLSR regression model for calculating rice biomass and N nutrition status was developed, and the final calibration and validation set of *R*^2^ reached 0.76 and 0.80, respectively [64].

Four machine learning models, PLSR, SVM, GBM, and RF, were compared to generate the best predictive models for NSC and N contents using UAV multispectral images and VIs. Our results showed that while there are gaps, using different models to predict different traits improved accuracy. In this study, the GBM prediction model produced the most reliable results; not only was the prediction accuracy of model prediction enhanced, but the validation and calibration sets were also more stable. Similarly, Barzin et al. [65] discovered better results using UAV multispectral data with the GBM model predicting an *R*^2^ value of 0.95 for N content in maize. Schucknecht et al. [66] examined 3 machine learning models, ML, RF, and GBM, and found that GBM had the highest accuracy in predicting plant N content and biomass, with an *R*^2^ of 0.47 and an RMSE of 0.45%.

These experiments show that the GBM model is reliable in predicting N content, with a greater prediction accuracy than the NSC content. The results of the PLSR analysis performed in our study were different from those obtained in earlier investigations. For instance, the PLSR model based on 5-band reflectance and 3 VIs attained the best accuracy in a prior study employing UAV multispectral imagery to forecast sugarcane canopy N concentration and irrigation levels (*R*^2^ = 0.79 and RMSE = 0.11%) [67]. Although the accuracy of the prediction model is higher than ours, it demonstrates the potential of UAV multispectral pictures in predicting leaf N and NSC contents. In addition, multiple studies have tested and compared the effectiveness of spectral indices and PLSR in predicting canopy N content of winter wheat, ultimately increasing the mean *R*^2^ of the PLSR model by 76.8% and 75.5%, respectively [27].

The SVM prediction model, similar to the PLSR prediction model, has higher accuracy in predicting NSC with an *R*^2^ of 0.75 on the calibration set; although the accuracy of these 2 prediction models is lower than the previous study results, the prediction of NSC content in this study is clearly reliable [68]. The RF model has the highest *R*^2^ on the calibration set of the NSC and N prediction models, consistent with earlier studies where RF beat PLSR [69]. The accuracy of the RF model on the prediction of N content is higher than the study by Moghimi et al. [70], who found that using aerial multispectral data with RF yielded an *R*^2^ of 0.55 in the prediction of N concentration in grape leaves. This study again illustrates the strong potential of RF models for NSC content. However, the difference is that the RF model for N and NSC contents predicts that *R*^2^ is too low on the validation set, which may be due to the small sample size, resulting in model instability and overfitting [71].

### Genetic variation in NSC and N contents and growth traits in slash pine

UAV technology has been successfully applied in agriculture and forest growth management. Reports have shown that using UAV remote sensing technologies to estimate the height of Scots pine yielded less error than field measurements [72]. The use of low-cost UAV imaging to estimate the height and canopy area of *Picea abies* L. [73], *Larix decidua* Mill produced favorable results [74]. However, there are few studies estimating NSC and N contents in canopy conifers by UAV multispectral technology due to the limited study background of slash pine. NSCs can provide plant growth with material and energy and participate in the synthesis of proteins and oil [75]. Our results showed that NSC and N contents in the canopy of slash pine varied in different months. Seasonal variations in NSC content are evident in our study. With the increase in new growth, canopy area, and turpentine synthesis beginning in March, a large number of nutrients must be consumed, and the NSC content gradually decreased. In October, tree growth tends to cease, and NSCs are once again stored in needles as NSCs [76]. Previous research has revealed that N influences the production and accumulation of chlorophyll and is an essential component of chlorophyll [77–79]. When the temperature rises in March, new shoots sprout, and photosynthesis is accelerated, resulting in a large number of new shoots and needles being produced. As the canopy area expands, the chlorophyll content and N content in the canopy increase substantially. This result also confirmed a strong positive correlation between chlorophyll and N [80]. Furthermore, our research validated the significant N concentration of newly developed branches [81]. In this study, the heritability and breeding values of physiological (NSC and N contents) traits of the slash pine family varied by month, allowing single qualities to be selected on the basis of different breeding requirements, as well as the association between traits. According to earlier related studies, in our study, the N content of *h*^2^ in March was as high as 0.5, and it had high breeding value in April, July, and November. The heritability and breeding values of NSC content were higher in July, and the heritability and breeding values of the canopy area and NSC content were higher in July, confirming the important role of NSCs in tree growth and metabolism [82]. Families can be chosen for a variety of breeding objectives. Comprehensive joint selection for all traits shows high variation in July, August, September, and October, and family selection can be conducted in these months, with the higher breeding value of all primarily dispersed in the first quadrant. Therefore, 5 families were chosen from these months, including 20, 6, 7, 9, and 15 (Fig. 8).

### Limitations and opportunities

This study represents one of the first studies estimating NSC and N contents in the canopy of slash pine using UAV multispectral technology and related information to estimate trait heritability and family breeding values over a single year. It serves as a useful proof of concept; however, the data collected only represent findings from a single year, meaning that the temporal variations among different years were not investigated. For future studies, repeated assessments are planned, especially investigating the variations in findings from drought and nondrought years.

## Conclusion

UAV multispectral technology combined with a machine learning algorithm was applied as a low-cost and high-throughput method to monitor the needle N and NSC contents of slash pine on a large-scale area in our study. This confirmed that UAV multispectral technology showed satisfactory and promising results in the prediction of needle N and NSC. In addition, UAV technology has been successfully adopted for tree breeding programs, which allows for long-term monitoring of tree growth traits and helps to understand genetic variation temporally. For the first time, the optimal timing of genetic selection for growth and canopy biochemical traits in slash pine were identified. Families with high breeding values of multiple traits were selected for further breeding use. In addition, the narrow-sense heritability and genetic variation across different years, which is also important, could be more convenient and reliable to be assessed using our methodology.

## Data Availability

The data mentioned in this paper are available on request from the corresponding author.
